# Anderson Insulators in Self-Assembled Gold Nanoparticles Thin Films: Single Electron Hopping between Charge Puddles Originated from Disorder

**DOI:** 10.3390/ma10060645

**Published:** 2017-06-12

**Authors:** Cheng-Wei Jiang, I-Chih Ni, Yun-Lien Hsieh, Shien-Der Tzeng, Cen-Shawn Wu, Watson Kuo

**Affiliations:** 1 Department of Physics, National Chung Hsing University, Taichung 402, Taiwan; unrealfish@gmail.com (C.-W.J.); yunlian13@gmail.com (Y.-L.H.); 2 Department of Physics, National Dong Hwa University, Hualien 974, Taiwan; ss311215@hotmail.com (I.-C.N.); sdtzeng@gms.ndhu.edu.tw (S.-D.T.); 3 Department of Physics, National Chang-Hua University of Education, Changhua 500, Taiwan; wucs@cc.ncue.edu.tw

**Keywords:** nanoparticles, metal-insulator transition, Anderson localization

## Abstract

The Anderson insulating states in Au nanoparticle assembly are identified and studied under the application of magnetic fields and gate voltages. When the inter-nanoparticle tunneling resistance is smaller than the quantum resistance, the system showing zero Mott gap can be insulating at very low temperature. In contrast to Mott insulators, Anderson insulators exhibit great negative magnetoresistance, inferring charge delocalization in a strong magnetic field. When probed by the electrodes spaced by ~200 nm, they also exhibit interesting gate-modulated current similar to the multi-dot single electron transistors. These results reveal the formation of charge puddles due to the interplay of disorder and quantum interference at low temperatures.

## 1. Introduction

The pioneer work by Mott pointed out the Coulomb interactions plays an important role in the electron conduction in a three-dimensional lattice, governed by the lattice constant. It is found that if the lattice constant is larger than a critical value, the system is an insulator at zero temperature; in the opposite case, it is a metal [1]. Later, Anderson proposed that in a random lattice, electron diffuses via quantum jumps between localized states, called charge puddles [2]. At low enough electron densities, no diffusion can take place and the system becomes so-called Anderson insulator (AI). Since then, the continuous metal-insulator transition (MIT) has been a subject of intensive study for decades [3]. However, the interplay between charge interaction and disorder leads to controversy over whether the electrons confined in a two-dimensional (2D) system can conduct at zero temperature. Initial theoretical investigation employing scaling theory concluded that disorder, no matter how weak, would trap charge carriers and convert any 2D metal into an insulator [4]. Against this view, Kravchenko reported an apparent MIT in 2D by measuring charge transport in silicon field-effect transistors by varying electron density [5]. This “plane mystery” attracts many theoretical and experimental efforts to unveil any exotic metallic phases [6].

Although MIT is observed in many kinds of materials, nanoparticle (NP) assemblies are considered to be an ideal platform for systematical study for the Mott–Hubbard transition in different dimensions [7,8]. The charge interaction, which quantified by a characteristic charging energy, is easily controlled by the NP size. The lattice constant can be well tuned by several means, such as the Langmuir method [9] and surface modification on NPs [10]. Indeed, in earlier experimental studies in self-assembled films of C*_n_*S_2_-linked Au NPs, electrical behaviors ranging from insulating to metallic-like were discovered. Following Mott’s prediction, the insulating property presents when the carbon chain length is greater than a critical value *n* = 5. This strong impact of the molecule linkage on the charge conduction originates from the exponential increase of tunneling resistance, *R_T_* with the number of carbon atoms: $R_{T}\propto \exp\left(\beta n\right)$. The tunneling decay constant per carbon atom *β* ~ 1.0 ± 0.1 for alkanedithiol molecular junctions [10,11]. In general, the Mott–Hubbard theory states that the charge conduction is mainly governed by the coupling strength, which is quantified by the dimensionless tunneling conductance *g* = *R_K_*/*R_T_*. *R_K_* = *h*/*e*^2^ ~ 25.8 kΩ is the quantum resistance. In the strong-coupling regime *g* >> 1, the NP assembly is metallic [12], but, in the opposite case *g* << 1, it becomes an insulator. Some experiments demonstrate the possibilities to control coupling strength by other means, such as NP coverage and film thickness [13,14].

Though successfully explaining the observed transition, the above picture is based on the regularity of the NP assembly, in which the randomness cannot simply be overlooked. There are at least three types of randomness: NP position and size, interparticle coupling, and random offset charges [15,16]. Theoretical studies indicate that Anderson localization exists when the disorder strength is large [17]. However, it was not reported until one decade after the first observation of Mott–Hubbard MIT [18], possibly because the temperature range in previous studies was too high to distinguish the AI from the metallic state. It turns out that AIs typically appear when the system is near the Mott–Hubbard MIT, displaying almost zero thermal activation energy for charge conduction. Nevertheless, they can be identified from the metallic phase with the dramatically upturning resistance and large negative magnetoresistance at very low temperatures [19]. As we have seen in 2D systems, the metallic phase could be overwhelmed by Anderson localization so a careful examination is needed. In this paper, we report measurement results on charge transport in the assembly of Au NPs with a few layers. Except controlling the length of molecular junctions, we take the advantage of electron beam exposure to reduce the junction barrier height. By these means, we could vary the coupling strength ranging from *g* = 10^−7^ to 10^2^. In particular, we present detailed results on AIs, including the temperature-dependent transport, magnetoresistance and device current modulated by a gate voltage. To begin with, let us review some important theoretical predictions on the Mott insulators (MIs).

### 1.1. Hubbard Bandgap and Mott Insulators

As a widely accepted model for electron conduction in NP assembly, Hubbard model features large Coulomb repulsion on the NP when electrons hop between them. The repulsion results in an activation energy for electron hopping, which can be quantified by the so-called charging energy given by the Abeles formula [20]:

(1) $$E_{C}=\frac{e^{2}}{8\pi \kappa_{m}\varepsilon_{0}}\times \frac{s}{r\left(s+r\right)},$$

because the NP is embedded in a large two- or three-dimensional network. Here, *r* is the NP radius and *s* is the interparticle spacing. *κ_m_* ~ 2.6 is the dielectric constant of the linkage molecule.

When there is interparticle charge tunneling, the charging energy deduced from electrostatics requires quantum correction because a smaller dwell time of a charge in an NP ensures a greater uncertainty in the energy. Such quantum fluctuations due to mobile charges become important when *R_T_* is comparable to *R_K_*. When *g* is not too large, the activation energy becomes

(2) $$E_{a}∼E_{C}\left(1-4\pi^{-1}zg\ln2\right),$$

in which *z* is the number of nearest neighbor NPs [12]. When *g* > 1, large quantum fluctuations dominate and turn the system into a metal, resulting in the Mott–Hubbard MIT at *R_T_* = *R_K_*. Owing to the existence of random charge offset in real NP assemblies, the energy cost for charge tunneling ranges between 0 and *E_C_*. Therefore the activation energy would be half of the value given by Equation (2) [21].

The charge transport in an MI is very similar to that in an intrinsic semiconductor. The temperature-dependent resistance *R*(*T*) follows a thermal activation law

(3) $$R\left(T\right)\propto \exp\left(\frac{T_{1}}{T}\right),$$

in which the characteristic temperature *T*_1_ = *E_a_*/*k_B_*.

### 1.2. The Cotunneling and Temperature-Dependent Resistance

We have mentioned that the above Hubbard model is insufficient to describe a disordered system. As first noted by Mott when studying disordered semiconductors, the electron hopping does not necessarily take place between nearest neighbors but may span a greater distance. Called variable-range hopping (VRH), this scenario describes a long-range hopping of balancing the charge hopping distance and energy cost [22]. In a large junction array like the NP assembly, a direct long-range hopping is impossible, but the cotunneling resembles VRH [23]. First, as a high order process, the cotunneling probability is exponentially suppressed by the number of involved junctions. Second, the cotunneling process may result in a long-range electron–hole pair, which is favorable in regarding electrostatic energy. There are two possible channels for the cotunneling process: elastic and inelastic channels. To see how VRH works, we first consider the charge hopping probability to an NP *j* spaced by a long distance *r_j_*, via the elastic cotunneling channel,

(4) $$\gamma_{j}\propto \exp\left(-\frac{r_{j}}{l_{el}}\right).$$

When the charging energy is short-ranged, the localization length is [24,25],

(5) $$l_{el}∼\frac{2a}{\ln\left(E\pi /g\delta \right)}.$$

Here, *a* = 2*r* + *s* is the average NP center–to-center distance. *E* is the average energy for short-range electron-hole excitation energies, roughly on the order of *E_a_*. δ is the electron energy level spacing in the NPs. With the same approach as Efros–Shklovskii (E–S) theory of VRH [26], the overall charge conduction should balance the charge hopping distance and energy cost, which arises from long-range Coulomb repulsion, $U∼e^{2}/4\pi \kappa \varepsilon_{0}r_{j}$. *κ* is the effective dielectric constant of the NP assembly. Therefore, the overall hopping probability should also include an Arrhenius factor, describing the thermal activation to overcome the energy *U*, and reads as

(6) $$\gamma_{j}\propto \exp\left(-\frac{r_{j}}{l_{el}}\right)\exp\left(-\frac{e^{2}}{4\pi \kappa \varepsilon_{0}r_{j}k_{B}T}\right).$$

Maximizing *γ_j_* by varying *r_j_*, one obtains a most probable hopping range,

(7) $$r^{*}∼\sqrt{\frac{l_{el}e^{2}}{4\pi \kappa \varepsilon_{0}k_{B}T}},$$

with the preferable hopping probability,

(8) $$\gamma^{*}\propto \exp\left(-\frac{2r^{*}}{l_{el}}\right)=\exp\left(-2\sqrt{\frac{e^{2}}{4\pi \kappa \varepsilon_{0}l_{el}k_{B}T}}\right).$$

In turn, the resistance obeys E–S VRH law

(9) $$R\left(T\right)\propto \frac{1}{\gamma^{*}}\propto \exp\sqrt{\frac{T_{2}}{T}},$$

where the characteristic temperature $k_{B}T_{2}∼2.8e^{2}/4\pi \kappa \varepsilon_{0}l_{el}$.

The calculation for inelastic cotunneling is more complicated. Apart from an Arrhenius factor suppressed by the energy difference between the final and initial states, the charge hopping probability can be enhanced by thermal fluctuation. The overall effect, when optimizing the probability by varying hopping distance, we also get the same result as Equation (9) but with a temperature-dependent localization length

(10) $$l_{in}∼\frac{2a}{\ln\left(E^{2}/16\pi T^{2}g\right)}.$$

However, as we will see later, its temperature dependence (~ln *T*) is much weaker than r^*^, (~*T*^−1/2^) so the resistance still follows the relation ln *R* ~ *T*^−1/2^ well. If comparing Equations (5) and (10), one finds that the inelastic cotunneling dominates over the elastic one when $l_{in}>l_{el}$ or ${k\;}_{B}T>\sim 0.1\sqrt{E\delta }$.

The charges may conduct via either thermal activation (Equation (3)) or cotunneling processes (Equation (9)). As a higher order process, the cotunneling typically has a small contribution to the charge transport, but its weaker temperature dependence makes it dominate over thermal activation at low temperatures. In short, when $T>>T_{cr}=T_{1}^{2}/T_{2}$, thermal activation mainly governs the charge transport, while, in the opposite limit, VRH becomes dominant.

## 2. Results and Discussion

### 2.1. Temperature Dependent Resistance

**Figure 1:**
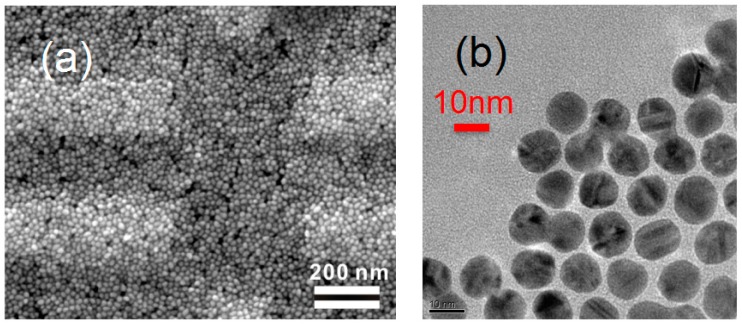
(**a**) the scanning electron micrograph of a 8-mercaptooctanoic acid (MOA) device; rectangles with a light color are the measurement electrodes; (**b**) the transmission electron microscopy image of the synthesized Au nanoparticles deposited on a Si_3_N_4_ membrane. Here, the capping molecules are 11-mercaptoundecanoic acid (MUA) molecules.

Figure 1a shows the multilayer Au NP film deposited on the substrate with premade electrodes. The gap between adjacent electrodes is 200 nm. The transmission electron microscopy image shown in Figure 1b reveals that the synthesized Au NPs are highly uniform in size, having an average diameter of 12 nm. Great variations in resistance *R* for devices with different surface modification molecules were found at room temperature. Enlisted in Table 1, the resistances show a high correlation to the interparticle distance *s*, which is estimated from *s*/nm = 0.54 + 0.12 *n* [27]. For most of the devices, the exponential dependence *R* ~ exp (*β_x_s*) are found, with *β_x_* ~ 11.8 nm^−1^.

**Figure 2:**
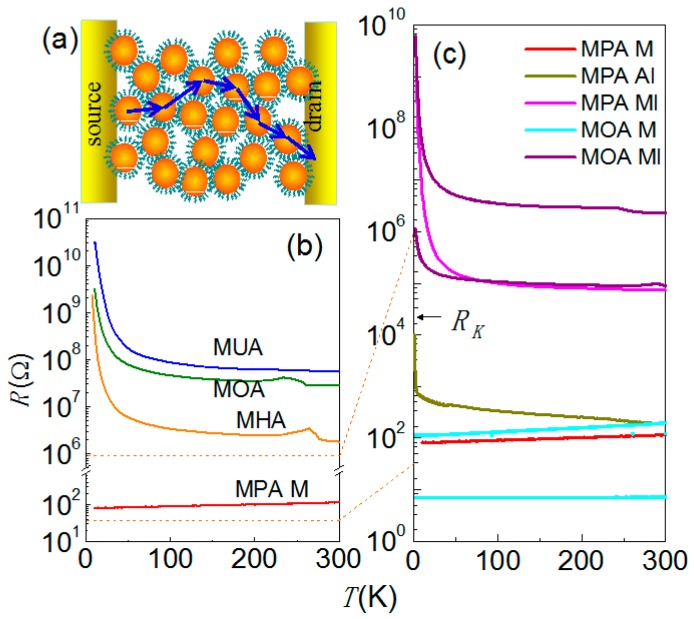
(**a**) the schematic of a two-terminal device; (**b**) the *R* vs. *T* curves for MUA, MOA, MHA and metallic MPA (MPA M) devices. The MPA M device shows metallic behavior while the others show insulating behavior; (**c**) *R*(*T*) curves for various MPA and e-beam exposed MOA devices. MPA devices may have versatile *R*(*T*) behaviors: when *R_RT_* > *R_K_*, the device is an insulator (MPA MI). When *R_RT_* < *R_K_*, it can be metallic (MPA M) or insulator (MPA AI) due to different disorder strength. The e-beam exposure may reduce the device *R_RT_* and turn a MOA device into a metal.

The temperature-dependent resistance, *R*(*T*), typically provides simple criteria for judging whether a device is metallic or insulating. As one can see in Figure 2b, a metallic behavior that the resistance increases linearly as temperature rises (*dR*/*dT* > 0) was found for Au NP films modified by short (MPA) carbon-chain molecules; an insulating behavior that the resistance decreased non-linearly was found for the AuNP films modified by long (MUA, MOA and MHA) carbon-chain molecules (*dR*/*dT* < 0). For MOA and MHA devices, a small resistance hump is found at the temperature around 235 K, which could be attributed to the contraction/expansion of residual water in the film. At low temperatures, these insulating devices exhibit non-linear current-voltage (*IV*) characteristics. These behaviors briefly signify an MIT by tuning the carbon number from *n* = 3 to *n* = 6 (*s* from 0.90 nm to 1.27 nm) [10,28].

Near the transition, the metallic and insulating behaviors require a closer inspection because disorder may give substantial impact. In particular, NP assemblies linked by MPA molecules may have versatile *R*(*T*) behaviors: although most MPA devices (referred to MPA M) are metallic that exhibit linear *IV* and *dR*/*dT* > 0, we could find exceptions, noted by MPA AI and MPA MI devices, showing insulating behavior. As illustrated in Figure 2c, the metallic or insulating behaviors of MPA devices are closely related to their room-temperature (RT) resistance, *R_RT_*: A high-*R_RT_* device tends to be insulator-like. Since the Au NP assemblies using our deposition scheme were not always closely packed and regular, *R_RT_* for a specific molecule modification varied from sample to sample. To clarify the role of *R_RT_* in MIT, we lowered the junction barrier height of insulating MOA devices by applying high dosage e-beam exposure. The exposure reduced the *R_RT_* of MOA devices at most five orders of magnitude and drove some devices across the critical point of MIT at *R_RT_* ~ *R_K_*. As one can see in Figure 2c, e-beam exposed MOA devices may exhibit metallic (MOA M) or insulating (MOA MI) behaviors, evidently relating to their *R_RT_*. In addition to the interparticle coupling strength, the disorder, which is hardly determined by *R_RT_*, serves as another parameter governing the MIT in these NP assemblies.

### 2.2. Mott Insulator and Cotunneling in Molecule Junctions 

**Figure 3:**
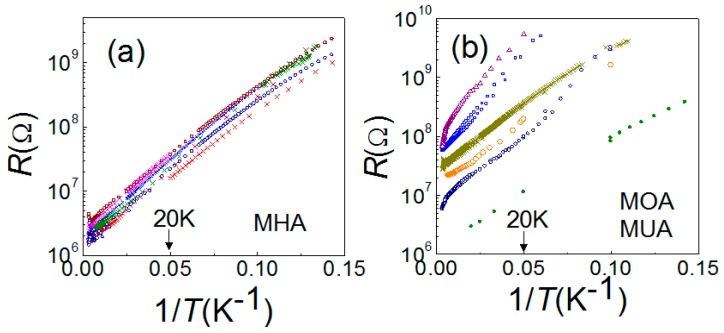
*R* vs. 1/*T* plots for insulating MHA (**a**), MOA and MUA (**b**) devices. Each color represents individual device. At higher temperatures, the resistance follows the thermal activation property, namely, ln *R* ~ *T*^−1^. There is a slight deviation near room temperature, suggesting effect from thermal expansion.

According to the theoretical prediction for MIs, the charge transport should follow the thermal activation law, Equation (3). It is easy to test the validity by plotting the device resistance *R* as a function of 1/*T* for insulating devices. As shown in Figure 3, MHA, MOA and MUA devices satisfy such a law when *T* > 20 K. The slope of this plot gives the characteristic temperature *T*_1_, a measure of Hubbard gap. Summarized in Table 1, *T*_1_ falls in the range between 40 and 80 K, except some devices with *R_RT_* < *R_K_*. As we have mentioned, it is plausible to assume that *R_RT_* is a good estimation of tunneling resistance in the theoretical model. Consequently, the insulating devices with *R_RT_* > *R_K_* receive negligible charge fluctuation so *T*_1_ ~ *E_C_*/*k_B_*, which is similar in our devices with the same NP size. According to Abeles formula, *E_C_*/*k_B_* ranges from 50 K to 75 K depending on the value of interparticle distance *s*. In contrast, the devices with *R_RT_* close to *R_K_* would have a much smaller *T*_1_, typically smaller than 10 K, because of the quantum correction. In an earlier work, we already presented how *T*_1_ depends on the tunneling resistance and found a good agreement with Equation (2) [19].

**Figure 4:**
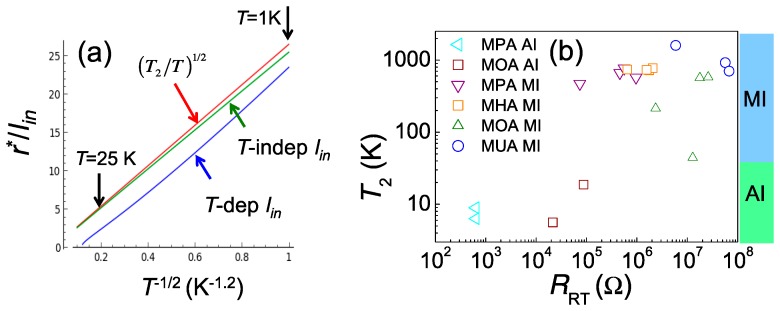
(**a**) hopping exponent (r^*^/*l_in_*) vs. *T*^−1/2^ plots for temperature-dependent *l_in_* using Equation (10) (blue), and temperature-independent *l_in_* = 0.1 *a* (green). The red curve is a function plot, (r^*^/*l_in_*) = (*T*_2_/*T*)^1/2^ with *T*_2_ = 700 K. The slopes of all curves are very similar; (**b**) a summary of *T*_2_ values for insulating devices. Clearly, the devices can be divided into two categories. Devices with *R_RT_* larger than 10^5^ Ω have *T*_2_ larger than ~ 100 K.

At lower temperatures, the disorder may result in the charge cotunneling in the molecule junctions, producing a transport resembling the E–S VRH. Before analyzing our data that is mostly measured at *T* > 2 K, we first judge that they are better explained by the inelastic channel, based on the fact that the elastic-inelastic crossover temperature $0.1\sqrt{E\delta }/k_{B}$ ~ 2 K with the level spacing δ ~ 0.1 meV and *E*/*k_B_*(~*T*_1_) = 50 K. Moreover, we should look if E-S VRH law is well obeyed with a temperature-dependent *l_in_*. Calculation on the hopping exponent (r^*^/*l_in_*) was performed by using parameters obtained in our experiments: *a* = 12 nm, *g* = 0.01, *E*/*k_B_*(~*T*_1_) = 50 K, *κ* = 21.5. The blue curve in Figure 4a shows temperature-dependent *l_in_* using Equation (10), while the green curve shows temperature-independent *l_in_* = 0.1 *a*. As a comparison, the function (r^*^/*l_in_*) = (*T*_2_/*T*)^1/2^ with *T*_2_ = 700 K is plotted with the red curve. Indeed, Equation (10) describes a slowly varying function of *T*, so the blue curve is highly linear in the temperature regime from 25 K to 1 K. In addition, all curves show similar slopes in this plot.

Figure 4b illustrates a summary of *T*_2_ values for insulating devices. One could see that *T*_2_ ~ 1000 K for devices with *R_RT_* > *R_K_*. As discussed in the previous section, a large *R_RT_* guarantees a weakly coupled NP network and a standard MI. As such, the cotunneling length *l_in_* ~ *a* according to Equation (10). An effective dielectric constant *κ* ~ 20 is much larger than that of the insulating matrix molecules *κ_m_*, owing to the charge screening effect from mobile charges within nearby NPs.

Devices having a lower *R_RT_* follow the E–S VRH law but with a much lower *T*_2_ value. The unusual low *T*_2_ could result from a huge *κ* and/or a very large *l_in_*. The later seems unreasonable in the scope of charge tunneling in weakly coupled NPs; however, these low *T*_2_ systems are on the threshold of strong coupling, so a new picture beyond inter-NP charge hopping is needed. If *κ* is unchanged, the cotunneling length is increased by two orders of magnitude, inferring the charge delocalized from a single NP but confined in a larger space *l_in_* ~ 10–100 *a*. Such a confinement can be argued to originate from Anderson localization due to quantum interference. To confirm this, one should investigate the magnetic response of these devices.

### 2.3. Magnetoresistance

**Figure 5:**
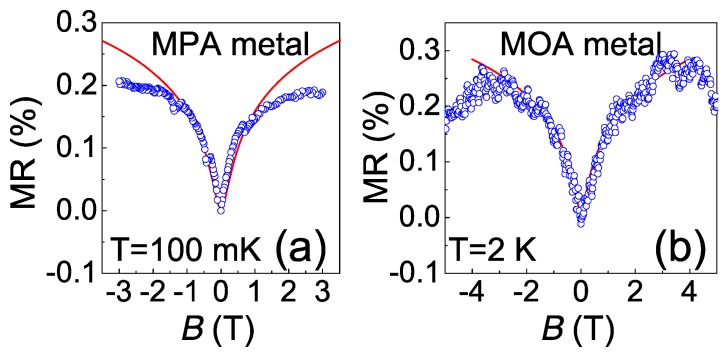
Positive MR in metallic devices. (**a**) an MPA metallic device in the parallel field at *T* ~ 100 mK; (**b**) an MOA metallic device in the perpendicular field at *T* = 2 K. Red curves are fitting results by using Equations (11) and (12).

It is beneficial to examine the magnetic response of the charge transport in metallic devices in the beginning. Figure 5a,b respectively illustrate the magnetoresistance (MR) of an MPA metal in the parallel field at *T* ~ 100 mK and a MOA metal in the perpendicular field at *T* = 2 K. Both cases show a positive MR saturated at a field about *B* ~ 3 T, and can be explained by the weak anti-localization. Owing to the strong spin-orbit interaction, weak anti-localization is commonly observed in metals with large atomic weight, such as Ag and Au. In NP assemblies, the electron may be scattered by the defects inside the NP (bulk scattering) and the molecule junctions. For metallic devices, NPs are strongly coupled and the electron wave function may extend across large numbers of NPs so the bulk scattering with large spin-orbit interaction is dominant.

The theory of weak anti-localization asserts the conductivity change in a magnetic field perpendicular (⊥) or parallel (//) to the film as [29]:

(11) $$\Delta \sigma_{⊥}≃-\frac{e^{2}}{h}\Psi \left(\frac{l_{H}^{2}}{2l_{e}l_{\phi }}+\frac{1}{2}\right)+\frac{e^{2}}{h}\ln\left(\frac{l_{H}^{2}}{2l_{e}l_{\phi }}\right),$$

(12) $$\Delta \sigma_{∥}≃-\frac{e^{2}}{h}\ln\left(1+\frac{t^{2}l_{e}l_{\phi }}{6l_{H}^{4}}\right),$$

in which *t* is the film thickness, $l_{H}=\sqrt{\hbar /eB}$ is the magnetic length, while *l_e_* and *l_φ_* are elastic and inelastic scattering lengths. Ψ is the digamma function. Figure 5a also presents the fitting result according to Equation (12) by using *t* = 30 nm, and a sheet resistivity of about 12 Ω. The characteristic length $\sqrt{l_{e}l_{\phi }}$ ~ 300 nm, which gives a minimal inelastic scattering *l_φ_* ~ 9 µm if one accounts for the upper bound of *l_e_* ~ 2*r* = 12 nm. In contrast, the fitting result for Figure 5b gives a characteristic length $\sqrt{l_{e}l_{\phi }}$ ~ 100 nm. The reduction of *l_e_* and *l_φ_* for the latter case is probably due to the electron–photon scattering at a higher temperature. Nevertheless, the MR indicates a large *l_φ_* and vigorous quantum inference for conduction electrons at *T* < 1 K.

**Figure 6:**
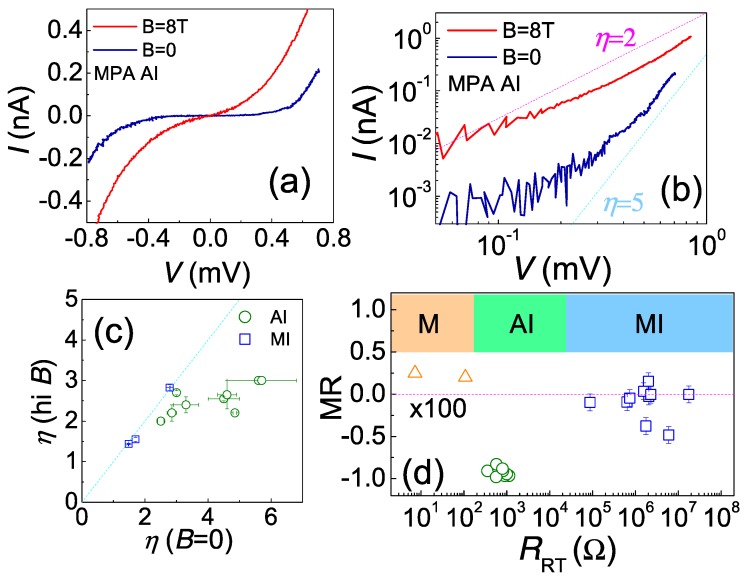
(**a**) The *IV* characteristics of an MPA AI device at *B* = 0 (blue curve) and *B* = 8 T (red curve) at 50 mK; (**b**) log-log plot of the *IV* curves in (**a**). The *IV* exponent *η* reduces from ~5 to ~2 when *B* is elevated from 0 to 8 T; (**c**) the change of *IV* exponent *η* from zero fields to the high field (MI at *B* = 9 T, AI at *B* = 8 T); (**d**) a summary of MR as a function of RT resistance. Metallic devices exhibit a small, typical 0.2% positive MR at *B* ~ 3T (*T* < 100 mK). MIs do not present magnetic response at the high field of *B* = 9 T (*T* > 2 K). For AIs, the zero bias resistance may be reduced as much as 10 times by a large field, *B* = 8 T (*T* < 100 mK).

Insulating devices may exhibit two distinct MR behaviors, which are closely related to their *T*_2_ and *R_RT_*. Devices having large *T*_2_ values are virtually not affected by the magnetic field. In contrast, extraordinary magnetic field responses were observed in devices having low *T*_2_ values (typical < 10 K). Figure 6a illustrates the *IV* characteristic of a low *T*_2_ MPA device at *B* = 0 and *B* = 8 T at *T* ~ 50 mK. In both cases, the *IV* presents sub-meV gap, of which the size clearly shrinks in the large field. When *V* > 0.05 mV, the non-linear *IV* curves follow a power law, $I\propto V^{\eta }$ as shown in Figure 6b. At zero field, the *IV* exponent *η* is ~5, while it reduces to ~2 in a large magnetic field. The quantitative change in *IV* curves for some insulators is best illustrated in Figure 6c, in which we summarize how the *IV* exponent *η* changes with the field. It is found that *η* can be reduced by one-half at most. A power law in *IV* curve is predicted by the cotunneling theory for weakly coupled NPs, and the exponent η=2j−1, where *j* is the number of junctions involved in the cotunneling process [30]. However, such a picture needs correction before applied to AIs, since the localization length for cotunneling may be very large, *l_in_*>> *a*.

Our non-magnetic devices should not present significant magnetic response unless the charge transport is strongly affected by quantum interference, which can be easily destroyed when a magnetic field is applied. Despite the positive MR in metals, we always find a negative MR for insulators, inferring that the charges are delocalized by the magnetic field. Therefore, it clearly shows that the charge gap strongly relies on the presence of quantum interference, following the description of Anderson localization. As such, these devices are considered as Anderson insulators. Additional evidence is the reduction of *η*, which relates to the numbers of junction involving in cotunneling in a large field. Two possibilities may result in this: the magnetic field weakens the charge–charge interaction, effectively reducing the most probable hopping range r^*^, or the field simply enlarges the inter-junction distance. For the former one, *η* for all kinds of insulator should be changed, which is not true for MIs. The later one is meaningful only for AIs, in which “inter-junction” distance is the size of charge puddles, not of NPs.

Figure 6d presents a summary of MR values measured at low temperatures (for MIs at *T* > 2 K and for others at *T* < 100 mK) in comparison to their RT resistances, *R_RT_*. Again, we can see a good correlation between MR and *R_RT_*: when *R_RT_* is smaller than ~100 Ω, the device presents a small positive MR, typical at the order of 0.2%, featuring the weak anti-localization in a disordered metal. When *R_RT_* is greater than ~10^5^ Ω, we see very small MR except for two special cases. When *R_RT_* is in between, the device would present large negative MR. In most cases, the resistance can be reduced for one to two orders of magnitude at *B* = 8 T. This infers that the magnetic response is a good indication for telling the difference between an MI and an AI.

**Figure 7:**
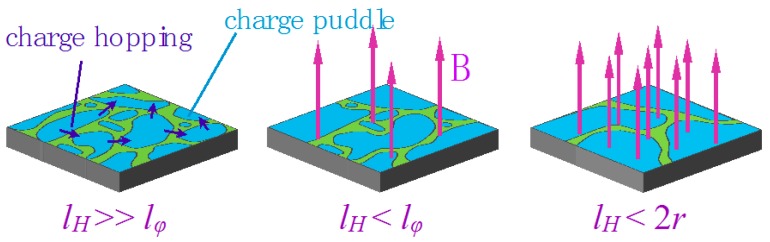
The formation of charge puddles in the AI and charge hopping between them. The red arrows show the perpendicular magnetic fields *B*. At the intermediate magnetic field *l_H_* < ~*l_φ_*, the size of the charge puddles becomes enlarged. When the field is so large that *l_H_* < 2*r*, the quantum interference is totally destroyed.

Here, we would like to comment on the important length scales for AIs, which is schematically illustrated in Figure 7. The localization length of AIs should not be larger than the electron inelastic scattering length and the magnetic length and has to be larger than the NP size. Therefore, we could set criteria for observing AIs in NP assemblies, min (*l_φ_*, *l_H_*) > 2*r*. To meet the criteria in our case, low temperature and low magnetic field—*B* ~ 0.1 T are essential. As the magnetic field increases, the charge puddles should be gradually enlarged, and an external gate voltage can be employed to probe the size change. We will discuss the gate-voltage modulation in the following section.

### 2.4. Single Electron Tunneling and Gate-Modulated Transport

**Figure 8:**
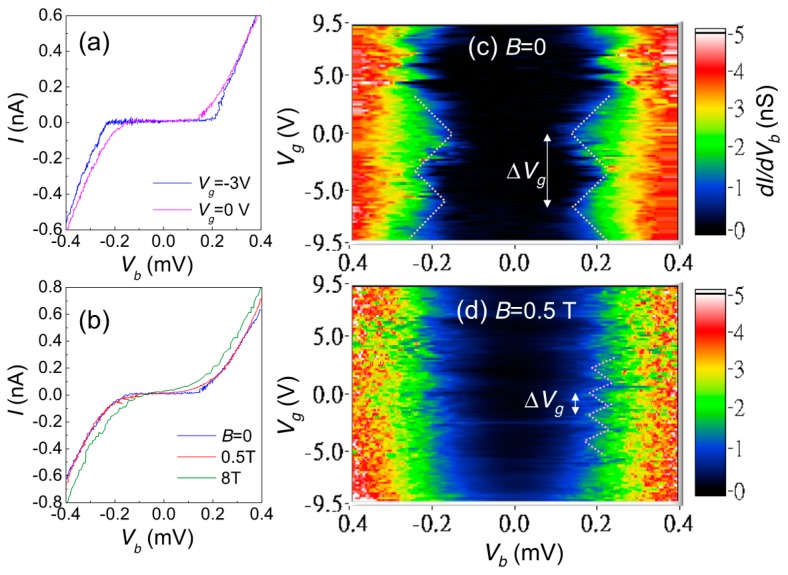
(**a**) the *IV_b_* characteristics for an MPA “single electron transistor (SET)” device with back-gate voltage *V_g_* = 0 V and −3.0 V, which respectively give the maximum and minimum current; (**b**) the *IV_b_* characteristics in various magnetic fields, *B* = 0 T, 0.5 T and 8 T at *V_g_* = 0 V. In a small magnetic field, the Coulomb gap is not affected much; (**c**) intensity plots of *dI/dV_b_* as a function of *V_g_* and *V_b_* in zero fields. The structure similar to Coulomb diamonds signifies single electron tunneling in the device. From the plot, one can clearly determine a gate-modulation period Δ*V_g_* ~ 6.5 V; (**d**) in the field *B* = 0.5 T, the gate-modulation period shrinks to Δ*V_g_* ~ 1.0 V.

At mK temperatures, MPA AIs present significant gate-modulated transport with the application of back-gate voltage. Because the Si substrate becomes insulating at low temperatures, the conducting gate electrode is ~ 0.5 mm far below the Au NP film. With such a distance, the current change is perceptible when applying a large gate voltage on ~1 V scale. Figure 8a shows *IV_b_* characteristics at *V_g_* = 0 and −3 V. Here, *V_b_* and *V_g_* respectively denote the source–drain bias voltage and gate voltage. When *V_g_* = −3 V, the threshold voltage for charge conduction ~0.22 mV, and when *V_g_*= 0 V, the threshold voltage decreases maximally by ~0.1 mV. Although the magnetic field also reduces the Coulomb gap, here the gate modulation is much smaller than the magnetic change. One can see this by comparing *IV_b_* characteristics in zero field, *B* = 0.5 T and *B* = 8 T, as illustrated in Figure 8b. When the field is small, *B* = 0.5 T, the Coulomb gap is almost unchanged, but as we will see later, such a small field already alters the gate modulation into a very different pattern from that at zero fields.

The gate modulation at low fields may display the pattern of a single electron transistor (SET). Figure 8c shows the dynamical conductance, *dI*/*dV_b_* as a function of *V_b_* and *V_g_* for such a typical device. Guided by the dot lines, we clearly see a regular modulation of the threshold voltage by *V_g_* with a period of Δ*V_g_* ~ 6.5 V, presenting the signature of “Coulomb diamond”. In addition to the main structure, modulations with a smaller period are also seen, though the period is not clear to estimate. In a small field 0.5 T, the gate modulation changes to a very different pattern as shown in Figure 8d: both the gate-modulated threshold voltage and the period shrinks in the small field. We note that, when the field is increased to ~0.6 T, no gate modulation could be observed.

The single electron transport sensitive to magnetic fields is usually related to spin-dependent transport, such as those observed in ferromagnetic SETs. However, spin-dependent transport cannot explain the transport in our non-magnetic devices. The only possible explanation is that the SET geometry, such as size, shape or tunnel junctions are field-dependent. Indeed, the period of gate modulation reduces from Δ*V_g_* = 6.5 V to 1.0 V, giving an increase of gate capacitance from *C_g_* = *e*/Δ*V_g_* = 25 zF to 100 zF when the field rises to 0.5 T. At the same time, the gate modulated threshold voltage also reduces from ~0.1 mV to ~0.05 mV, suggesting that the single island charging energy is halved and the island capacitance is enlarged from *C* = *e*^2^/2*E_C_* ~ 0.8 fF to 1.6 fF. Apparently, both increments of *C* and *C_g_* provide strong evidence of an enlarged SET island in the application of magnetic fields. In particular, the vanishing of gate modulation suggests that the SET island may be destroyed in large fields.

As we know that when the electronic system is in a low electron concentration and there is notable disorder, the charge is localized to form charge puddles. It is very likely that the SET signature arises from the single electron hopping between charge puddles. If there is accidentally only one charge puddle inside the gap between the source and drain electrodes, we may see the single electron hopping to-and-fro between the source/drain leads and the puddle. The electric potential of the puddle is altered by the gate voltage so standard and single period Coulomb diamonds can be observed. Unlike the island of an ordinary SET, which is made of a conductor, the charge puddle is not rigid and could be deformed under the influence of electromagnetic fields. In particular, the magnetic field breaks the time-reversal symmetry required for Anderson localization. As the field increases, charge puddles may merge into bigger ones and eventually grow into a very large, single one. Then, the system comes to a situation that inter-puddle hopping disappears and gate modulation becomes difficult to observe. Moreover, because the quantum interference is completely suppressed when *l_H_* < 2*r*, the field-sensitive gate-modulation cannot exist in a field higher than this limit.

**Figure 9:**
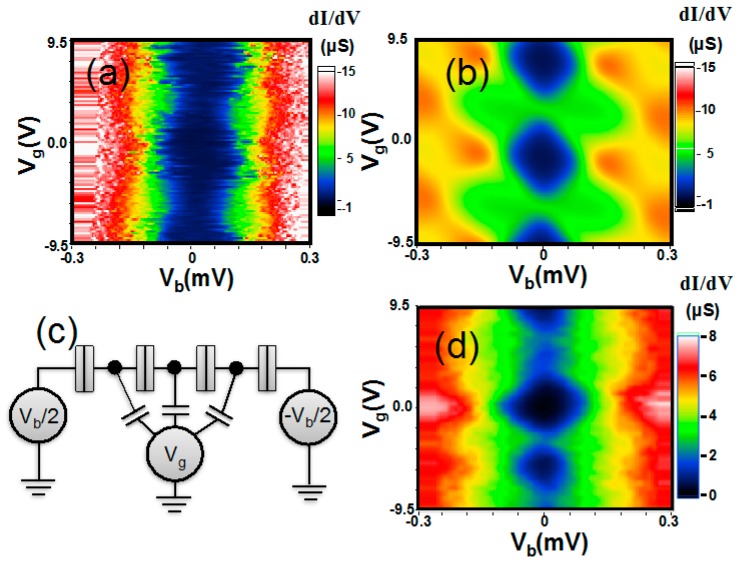
(**a**) the experimental data of dynamical conductance for a different MPA AI device; (**b**) the calculated dynamical conductance of a single-island SET circuit; (**c**,**d**) the assumed three-island circuit and calculated dynamical conductance.

To understand the single-electron tunneling phenomena demonstrated by the MPA AI devices, we performed current calculations based on solving master equations for charge states of metallic islands [31]. Prior to the calculation, important parameters were estimated from the size of the Coulomb diamond as shown in Figure 9a, which shows a uniform gate modulation period. As a first trial, we assumed a (single-island) SET circuit, and fine-tuned circuit parameters according to experimental *IV_b_* curves. The best *dI*/*dV_b_*-plot we could get is presented in Figure 9b, in which the junction parameters are *C_J_*_1_ + *C_J_*_2_ = 1.4 fF, *R_J_*_1_ + *R_J_*_2_ = 100 kΩ, and *C_J_*_1_/*C_J_*_2_ = *R_J_*_2_/*R_J_*_1_ = 1.2. However, the calculation result would give a zero-threshold condition at *V_g_* = *e*/2*C_g_*, against the experimental observations. Then, the multi-island circuits were assumed in further trials. By using a series combination of three islands presented in Figure 9c and identical junction parameters, *C_J_* = 1.0 fF, *R_J_* = 25 kΩ, identical island-to-ground capacitance *C_g_* = 25 zF, and randomly distributed charge offsets, 0.2 *e*, −0.2 *e* and 0 *e*, we could obtain a *dI*/*dV_b_*-plot as illustrated in Figure 9d. This multi-island circuit could more reasonably explain the structure observed in Figure 9a.

**Figure 10:**
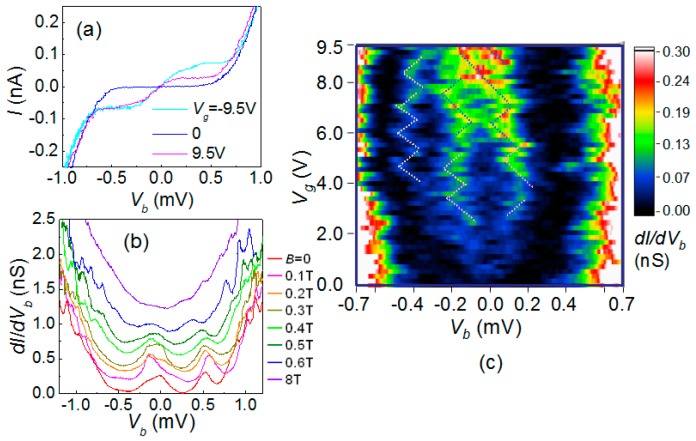
(**a**) *IV_b_* characteristics for an MPA “quantum dot” device with different back-gate voltage *V_g_* = 9.5 V, 0 V and −9.5 V, at which it presents staircase structure; (**b**) family of the dynamic conductance *dI*/*dV_b_* as a function of *V_b_* for another AI device at various magnetic fields. At low fields, *dI/dV_b_* shows resonance peaks. When the field is larger than 0.5 T, the resonance structures are smeared out; (**c**) intensity plots of *dI/dV_b_* as a function of *V_g_* and *V_b_*. As a guide to the eye, the dot lines illustrate the Coulomb diamond structure in this plot.

In addition to the SET-like charge transport, staircase-like *IV_b_* characteristics as illustrated in Figure 10a were observed in some MPA AI devices when *V_g_* was largely biased. Such staircase structures would result in resonance-like peaks in the dynamical conductance *dI*/*dV_b_* curve as in Figure 10b Again, we found that these resonance peaks become smeared as the magnetic field is elevated up to 0.5 T. When *B* > 0.6 T, the resonance peak and gate modulation disappear, together with the shrinkage of the Coulomb gap. The magnetic field destroying gate modulation is roughly the same as that for the SET-like devices, suggesting that they have the same origin.

Staircase-like *IV_b_* curves may have two possible origins: Coulomb staircase in a SET with asymmetric tunnel junctions, and the resonant tunneling in a quantum dot. The dynamical conductance as a function of *V_b_* and *V_g_* illustrated in Figure 10c is essential for the understanding of the underlying physics. To elucidate the origin, we calculated the device current based on an asymmetric SET circuit. By carefully choosing the model parameters, we may obtain some calculated *IV_b_* curve highly agreeing with the experimental data at a few *V_g_* values, but the calculated *dI*/*dV_b_*-plot shows structures of slanted Coulomb diamonds, a clear disagreement to the data shown in Figure 10c, where the resonance peaks move in two different directions that form symmetric diamonds. Therefore, the resonant tunneling likely explains the observed phenomenon. The discrete quantum levels may originate from the ultra low charge density and large wavelength comparable to the puddle size.

## 3. Materials and Methods

Au NPs were synthesized by reduction of AuCl_4_ by tannic acid and were sized 12 nm in diameter [32]. We used 4 different molecules to modify the Au NP surface: from short to long, 3-mercaptopropionic acid (MPA), 6-mercaptohexadecanoic acid (MHA), 8-mercaptooctanoic acid (MOA), and 11-mercaptoundecanoic acid (MUA). The longer molecules would introduce a longer interparticle spacing when Au NPs are assembled into a two-dimensional film. Au NP colloidal solution (typically 5 mL with concentration ~3 × 10^12^ cm^−3^) was added in a 30 mL centrifuge tube, together with the (3-aminopropyl)-trimethoxysilane(APTMS)- modified substrate laid on a support in the tube. After being centrifuged at 8500 *g* for 20 min, Au NPs were fully deposited on the SiO_2_/Si substrate, and we could directly get a multilayer Au NP film by gently pulling out the sample and drying it in the air. The thickness of the deposited Au NP film could be controlled by the total amount of Au NP in the solution and was further observed by the scanning electron microscopy. In this study, the typical thickness of the Au NP film is 2–3 NP layers. The details in deposition process, as well as thickness control, were described elsewhere [32]. Prior to NP deposition, 20 nm/50 nm Cr/Au electrodes were fabricated on the substrate by using e-beam or photolithography and lift-off technique.

MOA devices were further treated by high-dosage electron-beam (e-beam) exposure in a scanning electron microscope. The tunneling resistance does not only depend on the interparticle spacing but also on the tunneling barrier height. The high dosage e-beam bombardment may reduce the barrier height of the molecular junctions so as to greatly reduce *R_T_*. Electron microscopy imaging confirmed that the e-beam exposure did not change the NP network structure and the interparticle spacing *s*. *R_T_* for our samples was estimated from the monolayer sheet resistance at room temperature (RT). Because of the geometry of our device, the sheet resistance is on the same order of the device resistance. In addition, the e-beam bombardment enhances disorder strength of the exposed devices.

The temperature-dependent resistance measurements were performed in a physical property measurement system (Quantum Design Inc., San Diego, CA, USA) from RT to 2 K, and in a dilution refrigerator (Oxford Instruments, Oxfordshire, UK) from 1 K down to 40 mK.

## 4. Conclusions

In summary, our work revealed the important transport properties of an Anderson insulator, which is achievable by controlling the tunneling resistance *R_T_* ~ *R_K_* in an NP assembly with finite disorder. In such a system, the charge is mobile between the NPs but confined in charge puddles originated by the quantum interference due to multiple scattering. The charge puddle is fragile to the application of a magnetic field, which destroys the time-reversal symmetry. Because the size of charge puddles is on the order of the device dimension, it is possible to modulate charge transport hopping between charge puddles with a gate voltage, similar to a SET or a quantum dot. The breakdown of the charge puddle in large magnetic field results in a dramatic change of the gate-voltage modulation.

## Figures and Tables

**Table 1:** Important parameters of the Au nanoparticle devices.

Device	MUA	MOA	MHA	MPA MI	MOAe	MPA AI	MPA M
Carbon Number, *n*	11	8	6	3	8	3	3
*s* (nm)	1.88	1.51	1.27	0.90	1.51	0.90	0.90
*R_RT_* (Ω)	10^7^–10^9^	~10^7^	~10^6^	10^5^–10^6^	10^1^–10^7^	~10^3^	~10^3^
*T*_1_ (K)	43–80	40–52	45–56	32–49	17–50	<5	0
*T*_2_ (K)	700–1600	500–780	460–860	573–770	6–210	6–9	0
*l_in_* (nm)	2.4–4.9	4.2–4.7	6.8-7.7	8.3–11	-	-	-
*κ*	6–28	17–22	6–15	5–10	-	-	-
*E_C_* (meV)	11.0	9.3	8.1	6.0	-	-	-
*E_a_* (meV)	10.6–10.7	9.2	6.3–7.4	4.6–5.2	-	-	-

Note: MPA for 3-mercaptopropionic acid, MHA for 6-mercaptohexadecanoic acid, MOA for 8-mercaptooctanoic acid, and MUA for 11-mercaptoundecanoic acid. M for metal, MI for Mott insulator, and AI for Anderson insulator. MOAe for e-beam exposed devices and their properties range from metallic to insulating.

## References

[1] Mott N.F. (1949). The basis of the electron theory of metals, with special reference to the transition metals. Proc. Phys. Soc. Sect. A.

[2] Anderson P.W. (1958). Absence of diffusion in certain random lattices. Phys. Rev..

[3] Mott N.F. (1968). Metal-insulator transition. Rev. Mod. Phys..

[4] Abrahams E., Anderson P.W., Licciardello D.C., Ramakrishnan T.V. (1979). Scaling theory of localization: Absence of quantum diffusion in two dimensions. Phys. Rev. Lett..

[5] Kravchenko S.V., Kravchenko G.V., Furneaux J.E., Pudalov V.M., D’Iorio M. (1994). Possible metal-insulator transition at b = 0 in two dimensions. Phys. Rev. B.

[6] Smet J.H. (2007). Metal‚äìinsulator transition: A plane mystery. Nat. Phys..

[7] Stafford C., Das Sarma S. (1994). Collective Coulomb blockade in an array of quantum dots: A Mott–Hubbard approach. Phys. Rev. Lett..

[8] Collier C., Vossmeyer T., Heath J. (1998). Nanocrystal superlattices. Annu. Rev. Phys. Chem..

[9] Markovich G., Collier C.P., Heath J.R. (1998). Reversible metal-insulator transition in ordered metal nanocrystal monolayers observed by impedance spectroscopy. Phys. Rev. Lett..

[10] Zabet-Khosousi A., Trudeau P., Suganuma Y., Dhirani A., Statt B. (2006). Metal to insulator transition in films of molecularly linked gold nanoparticles. Phys. Rev. Lett..

[11] Xu B., Tao N.J. (2003). Measurement of single-molecule resistance by repeated formation of molecular junctions. Science.

[12] Beloborodov I.S., Efetov K.B., Lopatin A.V., Vinokur V.M. (2003). Transport properties of granular metals at low temperatures. Phys. Rev. Lett..

[13] Trudeau P.E., Orozco A., Kwan E., Dhirani A.A. (2002). Competitive transport and percolation in disordered arrays of molecularly-linked Au nanoparticles. J. Chem. Phys..

[14] Wessels J.M., Nothofer H.-G., Ford W.E., von Wrochem F., Scholz F., Vossmeyer T., Schroedter A., Weller H., Yasuda A. (2004). Optical and electrical properties of three-dimensional interlinked gold nanoparticle assemblies. JACS.

[15] Middleton A.A., Wingreen N.S. (1993). Collective transport in arrays of small metallic dots. Phys. Rev. Lett..

[16] Parthasarathy R., Lin X.-M., Jaeger H.M. (2001). Electronic transport in metal nanocrystal arrays: The effect of structural disorder on scaling behavior. Phys. Rev. Lett..

[17] Byczuk K., Hofstetter W., Vollhardt D. (2005). Mott–Hubbard transition versus Anderson localization in correlated electron systems with disorder. Phys. Rev. Lett..

[18] Jiang C.-W., Ni I.C., Tzeng S.-D., Kuo W. (2012). Anderson localization in strongly coupled gold-nanoparticle assemblies near the metal–insulator transition. Appl. Phys. Lett..

[19] Jiang C.-W., Ni I.C., Tzeng S.-D., Wu C.-S., Kuo W. (2014). Identification of Mott insulators and Anderson insulators in self-assembled gold nanoparticles thin films. Nanoscale.

[20] Abeles B. (1977). Effect of charging energy on superconductivity in granular metal films. Phys. Rev. B.

[21] Sugawara T., Minamoto M., Matsushita M.M., Nickels P., Komiyama S. (2008). Cotunneling current affected by spin-polarized wire molecules in networked gold nanoparticles. Phys. Rev. B.

[22] Mott N.F. (1969). Conduction in non-crystalline materials. Philos. Mag..

[23] Averin D.V., Odintsov A.A. (1989). Macroscopic quantum tunneling of the electric charge in small tunnel junctions. Phys. Lett. A.

[24] Feigel’man M.V., Ioselevich A.S. (2005). Variable-range cotunneling and conductivity of a granular metal. JETP Lett..

[25] Beloborodov I.S., Lopatin A.V., Vinokur V.M. (2005). Coulomb effects and hopping transport in granular metals. Phys. Rev. B.

[26] Efros A.L., Shklovskii B.I. (1975). Coulomb gap and low temperature conductivity of disordered systems. J. Phys. C Solid State Phys..

[27] Chen C.-F., Tzeng S.-D., Chen H.-Y., Lin K.-J., Gwo S. (2008). Tunable plasmonic response from alkanethiolate-stabilized gold nanoparticle superlattices: Evidence of near-field coupling. JACS.

[28] Collier C.P., Saykally R.J., Shiang J.J., Henrichs S.E., Heath J.R. (1997). Reversible tuning of silver quantum dot monolayers through the metal-insulator transition. Science.

[29] Markiewicz R., Rollins C. (1984). Localization and electron-interaction effects in a two-dimensional metal with strong spin-orbit scattering: Pd films. Phys. Rev. B.

[30] Dayen J.-F., Devid E., Kamalakar M.V., Golubev D., Guédon C., Faramarzi V., Doudin B., van der Molen S.J. (2013). Enhancing the molecular signature in molecule-nanoparticle networks via inelastic cotunneling. Adv. Mater..

[31] Grabert H., Devoret M. (1992). Single Charge Tunneling.

[32] Ni I.C., Yang S.C., Jiang C.W., Luo C.S., Kuo W., Lin K.J., Tzeng S.D. (2012). Formation mechanism, patterning, and physical properties of gold-nanoparticle films assembled by an interaction-controlled centrifugal method. J. Phys. Chem. C.

